# The Arginine/ADMA Ratio Is Related to the Prevention of Atherosclerotic Plaques in Hypercholesterolemic Rabbits When Giving a Combined Therapy with Atorvastatine and Arginine

**DOI:** 10.3390/ijms160612230

**Published:** 2015-05-29

**Authors:** Saskia J. H. Brinkmann, Elisabeth A. Wörner, Nikki Buijs, Milan Richir, Luc Cynober, Paul A. M. van Leeuwen, Rémy Couderc

**Affiliations:** 1 Department of Surgery, VU University Medical Center, 1081 HV Amsterdam, The Netherlands; E-Mails: sj.brinkmann@vumc.nl (S.J.H.B.); lisaworner@gmail.com (E.A.W.); n.buijs@vumc.nl (N.B.); m.richir@vumc.nl (M.R.); 2 Department of Surgery, Medical Center Alkmaar, 1815 JD Alkmaar, The Netherlands; 3 Laboratory of Nutrition Biology EA 4466, Faculty of Pharmacy, Paris Descartes University, 75006 Paris, France; E-Mail: luc.cynober@parisdescartes.fr; 4 Clinical Chemistry Department, Hopital Cochin et Hôtel-Dieu, AP-HP, 75014/75004 Paris, France; E-Mail: remy.couderc@trs.aphp.fr; 5 Clinical chemistry Laboratory, Armand Trousseau hospital, AP-HP, 75012 Paris, France

**Keywords:** atherosclerosis, arginine, ADMA, ratio, statins, nitric oxide, cholesterol

## Abstract

Supplementation with arginine in combination with atorvastatin is more efficient in reducing the size of an atherosclerotic plaque than treatment with a statin or arginine alone in homozygous Watanabe heritable hyperlipidemic (WHHL) rabbits. We evaluated the mechanism behind this feature by exploring the role of the arginine/asymmetric dimethylarginine (ADMA) ratio, which is the substrate and inhibitor of nitric oxide synthase (NOS) and thereby nitric oxide (NO), respectively. Methods: Rabbits were fed either an arginine diet (group A, *n* = 9), standard rabbit chow plus atorvastatin (group S, *n* = 8), standard rabbit chow plus an arginine diet with atorvastatin (group SA, *n* = 8) or standard rabbit chow (group C, *n* = 9) as control. Blood was sampled and the aorta was harvested for topographic and histological analysis. Plasma levels of arginine, ADMA, cholesterol and nitric oxide were determined and the arginine/ADMA ratio was calculated. Results: The decrease in ADMA levels over time was significantly correlated to fewer aortic lesions in the distal aorta and total aorta. The arginine/ADMA ratio was correlated to cholesterol levels and decrease in cholesterol levels over time in the SA group. A lower arginine/ADMA ratio was significantly correlated to lower NO levels in the S and C group. Discussion: A balance between arginine and ADMA is an important indicator in the prevention of the development of atherosclerotic plaques.

## 1. Introduction

Atherogenic risk factors, such as smoking, diabetes mellitus, hypertension and dyslipidemia, harm the endothelium and make it dysfunctional [1]. Endothelial nitric oxide (NO), released by the intact and healthy endothelium plays a very important role in the maintenance of vascular tone and structure [1]. NO has a number of intracellular effects that lead to vasorelaxation, endothelial regeneration, inhibition of leukocyte chemotaxis, and platelet adhesion. Therefore, decreased NO levels lead to endothelial dysfunction and this is an initial event in the development of atherosclerosis [2,3].

**Figure 1:**
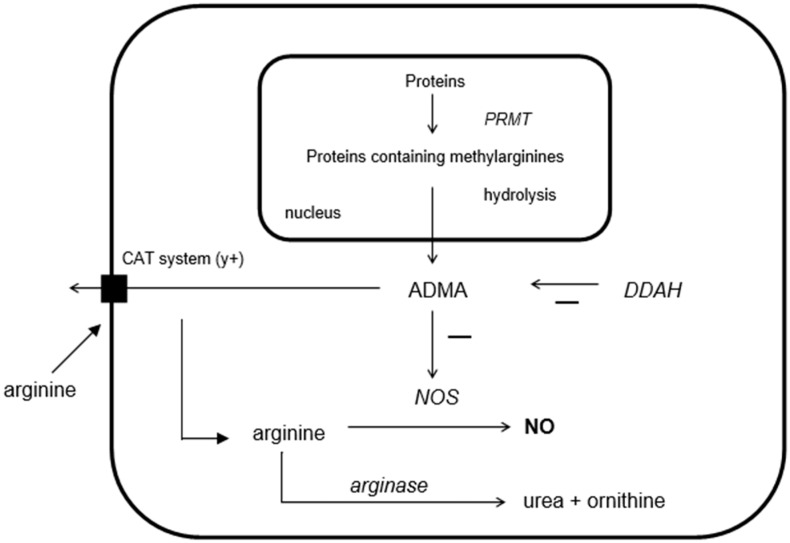
Schematic overview of the interactions between arginine, asymmetric dimethylarginine (ADMA), dimethylarginine dimethylaminohydrolase (DDAH), and nitric oxide synthase (NOS). PRMTs, protein arginine methyltransferases.

The formation of atherosclerosis can be overcome by increasing the synthesis of NO and providing its precursor arginine as a substrate to the endothelial cell [3]. Arginine is a semi-conditionally amino acid, which in its turn is the substrate for nitric oxide synthase (NOS) (see Figure 1). NOS converts arginine into NO and citrulline [4]. It has been shown that arginine supplementation increases NO availability, improves vascular responsiveness and reduces atherosclerosis in animals and patients [3,5]. Furthermore, endogenously produced inhibitors of the enzyme NOS, in particular asymmetric dimethylarginine (ADMA), also have an important role in NO metabolism [6,7]. ADMA and other methylarginines are continuously formed from intracellular proteolysis of methylated arginine residues in the nucleus of the cell, by enzymes called protein arginine methyltransferases (PRMTs) [8]. Whereas the structure of ADMA is similar to arginine, it competes with arginine for NOS binding, hereby blocking the formation of NO from arginine by NOS directly. NOS is mainly localized in the cell, thus the intracellular ADMA and arginine levels regulate NOS activity [9]. In addition, extracellular ADMA is an antagonist to extracellular arginine on cell membrane transporter level, whereas they are both transported into the cell via the cell membrane by the cationic amino acid transporter (y+ system), a high-affinity, Na^+^-independent transporter of the basic amino acids and therefore also compete with each other on this level [10]. Since ADMA competes with arginine for NOS and for cell transport via CAT-2, the bioavailability of NO depends on the balance between the two, the so-called arginine/ADMA ratio [11]. The arginine/ADMA ratio is an important indicator of NO bioavailability and therefore of the risk of formation of atherosclerotic plaques [11].

Statins are widely prescribed all over the world to treat hypercholesterolemia in current clinical practice. Statins are inhibitors of 3-hydroxy-3-methylglutaryl-coenzyme A reductase (HMG-CoA reductase), the rate limiting enzyme in the biosynthesis of cholesterol. Prospective clinical trials have demonstrated that reductions in low-density lipoprotein (LDL) cholesterol with statins decrease morbidity and mortality rates in particular in coronary artery disease and stroke [12,13]. A therapy that up-regulates NOS and subsequently NO through dietary manipulation of arginine, combined with a statin, would be the exquisite method to prevent and treat the formation of atherosclerotic plaques. Rasmusen *et al.* were the first to demonstrate that diet supplementation with arginine associated with atorvastatin was more efficient in reducing lesion size than treatment with arginine or statin alone in hypercholesterolemic rabbits [14]. The mechanism behind this feature remains unclear. The arginine/ADMA ratio is gaining more interest in the field of research as a potential marker of those of cardiovascular diseases [15,16,17]. Therefore, we hypothesized, as an ancillary study of Rasmusen *et al.*, that this ratio and changes in NO availability, could be the underlying factors of the positive effects of arginine combined with a statin on the development of atherosclerotic plaques in hypercholesterolemic rabbits. We investigated whether there was a (cor)relation between the ratio, levels of arginine and ADMA and the occurrence of atherosclerotic plaques as demonstrated in the study of Rasmusen *et al.* [14].

## 2. Results and Discussion

### 2.1. Results

#### 2.1.1. Effect of Treatment on l-Arginine Levels

At baseline (T0), mean plasma levels of arginine did not significantly differ between the groups. After eight weeks of treatment, arginine plasma levels increased significantly compared to T0 in the groups supplied with arginine (group A and SA, *p* < 0.001) (see Table 1).

#### 2.1.2. Effect of Treatment on ADMA and NO Levels

**Figure 2:**
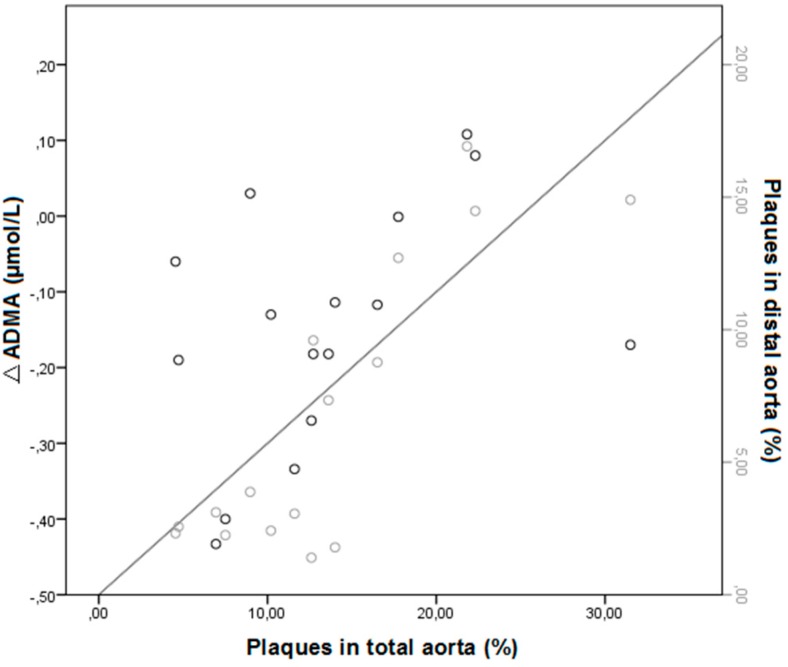
Pearson correlation between ∆ADMA (T0–T8, *n* = 4 per group) and aortic lesions in the distal aorta (*r* = 0.677, *p* = 0.01) and total aorta (*r* = 0.599, *p* = 0.03).

At T0 and T8, ADMA and NO levels did not significantly differ between groups. At the end of treatment (T8) ADMA levels decreased in all groups, but not significantly. The decrease in ADMA levels over time (T0–T8), when analyzing all groups together, showed to be significantly correlated to less aortic lesions in the distal aorta (*r* = 0.677, *p* = 0.01) and total aorta (*r* = 0.599, *p* = 0.03). Thus, the bigger the decrease in ADMA levels over time, the smaller the amount of arteriosclerotic lesions in the distal aorta (see Figure 2).

#### 2.1.3. Effect of Treatment on Arginine/ADMA Ratio and Relation with Other Parameters

**Figure 3:**
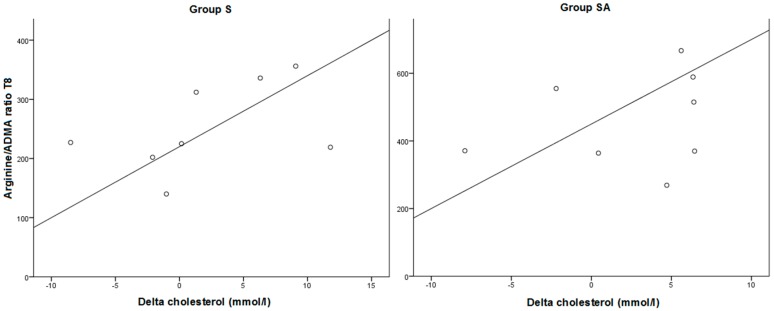
Pearson correlation between arginine/ADMA ratio and the difference in cholesterol levels over time in the statine (*n* = 8) and statine-arginine group (*n* = 8) (S: *r* = 0.461, SA: *r* = 0.699).

**Figure 4:**
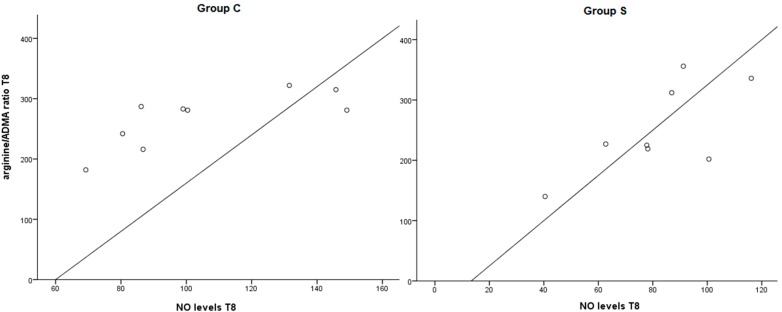
Pearson correlation between arginine/ADMA ratio T8 and NO levels at T8 in the statine (*n* = 8) and control group (*n* = 9) (S: *r* = 0.709, *p* = 0.049, C: *r* = 0.694, *p* = 0.056).

At T0, no significant difference between groups was found in arginine/ADMA ratio levels. The ratio was significantly increased at T8 in group A and SA (*p* < 0.05). A Pearson’s correlation test revealed the correlation between the arginine/ADMA ratio and cholesterol levels at T8, most pronounced in the SA group (*r* = −0.462). The arginine/ADMA ratio and cholesterol levels at T8 correlated positively (*r* = 0.279) in group A. In addition, the decrease in cholesterol over time was strongly correlated to the arginine/ADMA ratio in the S and SA group (S: *r* = 0.461, SA: 0.699) (see Figure 3). A lower arginine/ADMA ratio was significantly correlated to lower NO levels in the S and C group (S: *r* = 0.709, *p* = 0.049, C: *r* = 0.697, *p* = 0.056) (see Figure 4).

**Table 1:** Effect of different treatments on arginine levels, ADMA levels, arginine/ADMA ratio, and atherosclerotic lesions in the aorta.

Group	A (*n* = 9)			S (*n* = 8)			SA (*n* = 8)			C (*n* = 9)	
	Mean	SEM		Mean	SEM		Mean	SEM		Mean	SEM
Plasma levels l-arginine (µmol/L)											
T0	463	39		367	33		371	13		460	51
T8	388 ^a^	47		243 ^b^	28		476 ^a^	58		265 ^b^	14
Plasma levels ADMA (µmol/L)											
T0 ^∞^	1.14	0.11		1.02	0.03		1.15	0.05		1.11	0.05
T8	0.88	0.05		0.97	0.05		1.03	0.05		0.99	0.03
Arginine/ADMA ratio											
T0 ^∞^	347	19		304	42		317	23		374	43
T8	442 ^a^	46		252 ^b^	26		476 ^a^	54		261 ^b^	16
NO levels											
T0	110	20		88	9		103	10		108	16
T8	156	35		82	8		104	10		105	10
Total lesions in aorta (%)											
–	15.1	3.2		12.2	1.4		9.0	1.1		13.8	1.6
Distal lesions in aorta (%)											
–	9.8	2.6		5.4	1.3		3.0	0.7		6.8	1.3

Mean values with their standard errors. A, l-arginine group; S, statin group; SA, statin-l-arginine group; C, control group. T0, at the beginningof treatment; T8, after 8 weeks of treatment; NO, nitric oxide. ^a,b^ mean values within a row with different superscript letters are significantly different (*p* < 0.05) (independent samples test). Data about plasma arginine and NO are adapted from [14]. ^∞^ these plasma levels are based on *n* = 4 per group.

### 2.2. Discussion

The purpose of the present study was to determine the contribution of the arginine/ADMA ratio in the explanation of the positive effect from the combined therapy of arginine and a statin in the prevention of atherosclerosis as we reported previously [14]. In the present part of the study, we showed that arginine/ADMA ratio has a correlation to cholesterol, development of plaques and levels of NO in this model and could be a sensitive marker in the prevention of atherosclerosis by arginine and statin.

The arginine/ADMA ratio is gaining more interest in the field of research as a potential marker of cardiovascular diseases [15,16,17]. It is well-known that arginine is an important mediator in vascular flow and the integrity of the vascular wall by being the substrate of NO. Since ADMA inhibits NO production by competing with arginine for NOS binding, the net amount of NO production is indicated by the ratio between substrate and inhibitor, the arginine/ADMA ratio. Endothelium-derived NO plays a central role in normal vascular homeostasis. Impaired NO bioavailability causes endothelial dysfunction and not only contributes to the initiation and progression of atherosclerosis but is also associated with long-term risk of cardiovascular events [18]. NO is essential for flow-mediated dilatation. Richir *et al.* demonstrated that low arginine plasma levels in combination with high ADMA plasma levels deteriorates systemic hemodynamics and reduces blood flow through the kidney and spleen and liver [19].

Supplementation of arginine in a diet or intravenously, may contribute to higher levels of NO and maybe a nullification of the detrimental effects of ADMA. It was shown that dietary l-arginine reduces the progression of atherosclerosis and improves endothelium-dependent vasodilatation in cholesterol-fed rabbits [5,20,21,22]. Oral supplementation of l-arginine in hypercholesterolemic patients increases endothelial dependent vasodilatation in forearm conduit arteries [20,23]. Although l-arginine deficiency has never been documented in hypercholesterolemia, it seems that NO bioavailability is partly restored after l-arginine administration in hypercholesterolemic subjects [11,24]. Supplementation of dietary l-arginine can increase NO-mediated bloodflow [25,26]. In our study, arginine supplementation caused a two-fold increase in plasma arginine, which is consistent with previous studies [22,23].

However, just providing a substrate for NOS by supplementation of arginine cannot explain the positive effects on vasculature. All cells produce ADMA, the inhibitor of NO. It was shown that ADMA directly affects the integrity and function of vasculature itself by damaging the endothelial gap junction function, induction of smooth muscle cell migration, foam cell formation and apoptosis of smooth muscle cells and endothelial cells [27]. This was confirmed in our study setting in which we demonstrated that a decrease in ADMA levels over time showed to be significantly correlated to less aortic lesions in the distal aorta and total aorta. Thus, decreased ADMA levels may prevent the development of arteriosclerotic lesions in the aorta. It was shown that levels of ADMA are high in hypercholesterolemia, whereas levels of arginine have been found to be in the normal range [28]. This causes a shift in the arginine/ADMA ratio and results in diminished NOS activity and subsequently less NO bioavailability. This implicates the importance of the ratio above the sole levels of arginine in plasma. In the current study, lower levels of NO in the groups not supplied with arginine and/or statin, were correlated to the arginine/ADMA ratio. This confirms that a low arginine/ADMA low ratio deteriorates NO metabolism and therefore vascular flow in hypercholesterolemic rabbits.

The endothelial cell transporter that facilitates uptake of both arginine and ADMA is cationic amino transporter 1 (CAT1). ADMA inhibits not only NOS, but also this CAT transporter, that mediates cellular uptake of arginine. However, in normal physiological concentrations, ADMA cannot impair the CAT1-mediated transport of arginine [9,29]. Conversely, high (but still physiological) concentrations of arginine can inhibit CAT1-mediated cellular uptake of ADMA [20].

Rasmusen *et al.* confirmed the positive effects of statins on atherosclerotic plaques, because lower cholesterol levels after 8 weeks of treatment correlated significantly to less aortic lesions in the total and distal aorta [14]. In the present ancillary study, we revealed a positive correlation between the arginine/ADMA ratio and decrease of cholesterol levels over time and at the end of the treatment, primarily in the SA group. So when the ratio increases, cholesterol levels decrease, or inversely. In addition, a decrease in ADMA levels over time was significantly correlated to less aortic lesions in the distal aorta and total aorta. Statins are inhibitors of 3-hydroxy-3-methylglutaryl-coenzyme A reductase (HMG-CoA reductase) and reduce the amount of circulating LDL-cholesterol. Currently, seven types of statins are available: atorvastatin, fluvastatin, lovastatin, pitavastatin, pravastatin, rosuvastatin and simvastatin. Besides lowering levels of circulating LDL-cholesterol, statins exhibit different effects on NO-mediators. One study found that treatment with statins elevates levels of DDAH, which metabolizes ADMA [30]. Moreover, statins have shown to be able to decrease plasma levels of ADMA in different types of study settings [31,32,33,34,35,36]. Atorvastatin specifically is demonstrated to increase levels of DDAH in rats and humans and thereby to decrease plasma ADMA levels [37,38,39]. However, the effect of statins on ADMA concentrations is ambiguous, whereas some studies found that statins had no effect on ADMA levels in hypercholesterolemic subjects [40,41,42,43,44]. However, other intriguing experimental data have shown that statins exhibits pleiotropic effects beyond their lipid-lowering actions, including up-regulation of eNOS and thereby enhancement of endothelial NO production, inhibition of smooth muscle proliferation and anti-inflammatory and anti-oxidative actions [45,46]. Therefore, the exact combination of statins and arginine supplementation could have caused the correlation between the arginine/ADMA ratio and the decrease in cholesterol over time. Moreover, we did not find a correlation of arginine and ADMA alone with levels of cholesterol, so the combination of the two seems to determine the level of cholesterol in the arteries. This is supported by recently published data in which the arginine/ADMA ratio seemed to be a sensitive marker in the in the progression of atherosclerosis in means of intima-media thickness, rather than arginine or ADMA alone [16].

The limitation of the present study needs to be addressed. The number of Watanabe rabbits was relatively small in order to be a representative group. In order to transfer the effects measured to a clinical benefit, it needs to be researched in a clinical trial. We also suggest for upcoming studies to measure NOS and ADMA levels in response to the combination of statin and arginine in the vascular wall.

## 3. Experimental Section

This study is based on the results and dataset from the study of Rasmusen *et al.* [14].

### 3.1. Treatment of Animals

Thirty-four six-week-old homozygous Watanabe heritable hyperlipidaemic rabbits were assigned to one of four treatment groups: during 8 weeks, rabbits in the l-arginine group (group A; *n* = 9) were fed by a 1.5% l-arginine in 1 g/kg of bodyweight/d chow diet. This diet contained 16% crude protein, 3.2% fat, 49.3% carbohydrates, 13.6% fibre and 8.03 MJ gross energy/kg. Details of feeding regime were as described by Rasmusen *et al.* [14]. Arginine content in the diet was 0.98%. The statin group (group S; *n* = 8) consisted of rabbits receiving standard rabbit chow diet plus 2.5 mg/kg/d of atorvastatin in their drinking water [47]. In the l-arginine plus statin group (group SA; *n* = 8), rabbits received the l-arginine enriched rabbit chow together with atorvastatin in their drinking water. The control group (group C; *n* = 9) was fed with standard rabbit chow. Blood samples were collected at the vein of the ear of the rabbit into sodium heparinate (Sanofi, Winthrop Industry, Gentilly, France) and immediately centrifuged at +4 °C for 15 min at 4500× *g*. Blood samples were taken at the beginning of the treatment (T0) and at the end of the treatment period after eight weeks (T8). Due to ethical reasons and to prevent a hypovolemic situation, we were only allowed to take a limited amount of volume at T0. Because the inter- and intra essay variations are the smallest for ADMA, the number of plasma ADMA levels at T0 are determined in 4 rabbits per group.

Animal care and experimentation and the Council of Europe Guidelines agreed with the rules of the laboratory of Nutrition Biology of the Descartes University, Paris, France. Animal care and experimentation complied with the rules of our institution and with Council of Europe Guidelines. Chantal Martin (No. 75-10) and Christophe Moinard (No. 75-522) are licensed to conduct experimental studies on living animals, and we had received approval for the use of animal facilities (agreement No. A75-06-02, February 2006, Veterinary Service Directorate, Prefecture de Police de Paris, France).

### 3.2. Anatomy and Histological Analysis

For topographic and histological analysis the full length of the cervico-thoracic region of the aorta of the rabbits were obtained by Rasmusen *et al.* [14]. In brief, the areas of lesions were determined by using NIH Scion Image Software (Scion, Frederick, MD, USA). The areas were measured three times. The aorta was then prepared to 2 mm-thick fragments transversely cut into four segments (aortic arch, thoracic aorta, abdominal aorta and bifurcation), which were classified into distal lesions and lesions of the total aorta.

### 3.3. Biochemical Analysis

The concentrations of ADMA were determined simultaneously by high-performance liquid chromatography (HPLC). Plasma l-arginine levels were assessed on a JEOL automated amino acid analyzer (Tokyo, Japan) using ion-exchange chromatography [48]. The l-arginine over ADMA ratio was calculated. At T0, the arginine/ADMA ratio was only calculated from the rabbits of which ADMA was determined in plasma (*n* = 4 per group). To indicate the total NO production the products of NO, nitrite and nitrate, were measured in plasma as described in the Griess method [49]. Plasma total cholesterol was determined by routine enzymatic method on a Hitachi 911 analyzer (Roche, Meylan, France) [50].

### 3.4. Statistical Analysis

Results were tested upon distribution using Kolmogorov-Smirnov Test and QQ-plots. When normally distributed, results are presented in mean ± standard error of the mean (SEM) and in median and interquartile range when data were not normally distributed. The Student’s *t*-test or Mann Whitney U (according to data distribution) test was used to determine significant differences in arginine and ADMA concentrations between groups. The one-sample *t*-test was used to test whether the values were significantly different from the beginning of the treatment. Correlations between the continuous variables were assessed by using bivariate analysis to estimate the Pearson’s coefficient. SPSS for Windows software was used to perform statistical analysis. *p* < 0.05 was considered to indicate a significant difference.

## 4. Conclusions

In conclusion, supplementation of both arginine and a statin in hypercholesterolemic rabbits induces a high arginine/ADMA ratio and this is correlated to lower cholesterol levels after 8 weeks of treatment. The decrease in ADMA levels over time correlated to less aortic lesions in the distal aorta and total aorta. When hypercholesterolemic rabbits were not fed with arginine, a lower arginine/ADMA ratio was significantly correlated to lower NO levels. These results support the hypothesis that a balance between arginine and ADMA, being the substrate and inhibitor of NOS respectively, might contribute to better understanding of the development of atherosclerotic plaques. Further research on the combination of statins and l-arginine, preferably in clinical trials, is necessary to confirm our findings and translate them to a clinical setting.

## References

[1] Tousoulis D., Kampoli A.M., Tentolouris C., Papageorgiou N., Stefanadis C. (2012). The role of nitric oxide on endothelial function. Curr. Vasc. Pharmacol..

[2] Dias R.G., Negrao C.E., Krieger M.H. (2011). Nitric oxide and the cardiovascular system: Cell activation, vascular reactivity and genetic variant. Arq. Bras. Cardiol..

[3] Napoli C., de Nigris F., Williams-Ignarro S., Pignalosa O., Sica V., Ignarro L.J. (2006). Nitric oxide and atherosclerosis: An update. Nitric Oxide.

[4] Morris S.M. (2007). Arginine metabolism: Boundaries of our knowledge. J. Nutr..

[5] Boger R.H., Bode-Boger S.M., Brandes R.P., Phivthong-Ngam L., Böhme M., Nafe R., Mügge A., Frölich J.C. (1997). Dietary l-arginine reduces the progression of atherosclerosis in cholesterol-fed rabbits: Comparison with lovastatin. Circulation.

[6] Vallance P., Leone A., Calver A., Collier J., Moncada S. (1992). Endogenous dimethylarginine as an inhibitor of nitric oxide synthesis. J. Cardiovasc. Pharmacol..

[7] Vallance P., Leiper J. (2004). Cardiovascular biology of the asymmetric dimethylarginine: Dimethylarginine dimethylaminohydrolase pathway. Arterioscler. Thromb. Vasc. Biol..

[8] Teerlink T. (2005). ADMA metabolism and clearance. Vasc. Med..

[9] Blackwell S. (2010). The biochemistry, measurement and current clinical significance of asymmetric dimethylarginine. Ann. Clin. Biochem..

[10] Brinkmann S.J., de Boer M.C., Buijs N., van Leeuwen P.A. (2014). Asymmetric dimethylarginine and critical illness. Curr. Opin. Clin. Nutr. Metab. Care.

[11] Bode-Boger S.M., Boger R.H., Kienke S., Junker W., Frolich J.C. (1996). Elevated l-arginine/dimethylarginine ratio contributes to enhanced systemic NO production by dietary l-arginine in hypercholesterolemic rabbits. Biochem. Biophys. Res. Commun..

[12] Mihaylova B., Emberson J., Blackwell L., Keech A., Simes J., Barnes E.H., Voysey M., Gray A., Collins R., Baigent C. (2012). The effects of lowering LDL cholesterol with statin therapy in people at low risk of vascular disease: Meta-analysis of individual data from 27 randomised trials. Lancet.

[13] Baigent C., Blackwell L., Emberson J., Holland L.E., Reith C., Bhala N., Peto R., Barnes E.H., Keech A., Simes J. (2010). Efficacy and safety of more intensive lowering of LDL cholesterol: A meta-analysis of data from 170,000 participants in 26 randomised trials. Lancet.

[14] Rasmusen C., Moinard C., Martin C., Tricottet V., Cynober L., Couderc R. (2007). l-Arginine plus atorvastatin for prevention of atheroma formation in genetically hypercholesterolaemic rabbits. Br. J. Nutr..

[15] Bode-Boger S.M., Scalera F., Ignarro L.J. (2007). The l-arginine paradox: Importance of the l-arginine/asymmetrical dimethylarginine ratio. Pharmacol. Ther..

[16] Notsu Y., Yano S., Shibata H., Nagai A., Nabika T. (2014). Plasma arginine/ADMA ratio as a sensitive risk marker for atherosclerosis: Shimane CoHRE study. Atherosclerosis.

[17] Visser M., Vermeulen M.A., Richir M.C., Teerlink T., Houdijk A.P., Kostense P.J., Wisselink W., de Mol B.A., van Leeuwen P.A., Oudemans-van Straaten H.M. (2012). Imbalance of arginine and asymmetric dimethylarginine is associated with markers of circulatory failure, organ failure and mortality in shock patients. Br. J. Nutr..

[18] Lu T.M., Ding Y.A., Charng M.J., Lin S.J. (2003). Asymmetrical dimethylarginine: A novel risk factor for coronary artery disease. Clin. Cardiol..

[19] Richir M.C., van Lambalgen A.A., Teerlink T., Wisselink W., Bloemena E., Prins H.A., de Vries T.P., van Leeuwen P.A. (2009). Low arginine/asymmetric dimethylarginine ratio deteriorates systemic hemodynamics and organ blood flow in a rat model. Crit. Care Med..

[20] Clarkson P., Adams M.R., Powe A.J., Donald A.E., McCredie R., Robinson J., McCarthy S.N., Keech A., Celermajer D.S., Deanfield J.E. (1996). Oral l-arginine improves endothelium-dependent dilation in hypercholesterolemic young adults. J. Clin. Investig..

[21] Boger R.H., Bode-Boger S.M., Mugge A., Kienke S., Brandes R., Dwenger A., Frölich J.C. (1995). Supplementation of hypercholesterolaemic rabbits with l-arginine reduces the vascular release of superoxide anions and restores NO production. Atherosclerosis.

[22] Cooke J.P., Singer A.H., Tsao P., Zera P., Rowan R.A., Billingham M.E. (1992). Antiatherogenic effects of l-arginine in the hypercholesterolemic rabbit. J. Clin. Investig..

[23] Cooke J.P., Dzau J., Creager A. (1991). Endothelial dysfunction in hypercholesterolemia is corrected by l-arginine. Basic Res. Cardiol..

[24] Boger R.H., Bode-Boger S.M., Szuba A., Tsao P.S, Chan J.R., Tangphao O., Blaschke T.F., Cooke J.P. (1998). Asymmetric dimethylarginine (ADMA): A novel risk factor for endothelial dysfunction: Its role in hypercholesterolemia. Circulation.

[25] Bode-Boger S.M., Muke J., Surdacki A., Brabant G., Boger R.H., Frolich J.C. (2003). Oral l-arginine improves endothelial function in healthy individuals older than 70 years. Vasc. Med..

[26] Piatti P.M., Monti L.D., Valsecchi G., Magni F., Setola E., Marchesi F., Galli-Kienle M., Pozza G., Alberti K.G. (2001). Long-term oral l-arginine administration improves peripheral and hepatic insulin sensitivity in type 2 diabetic patients. Diabetes Care.

[27] Chen S., Li N., Deb-Chatterji M., Dong Q., Kielstein J.T., Weissenborn K., Worthmann H. (2012). Asymmetric dimethyarginine as marker and mediator in ischemic stroke. Int. J. Mol. Sci..

[28] Boger R.H., Bode-Boger S.M., Sydow K., Heistad D.D., Lentz S.R. (2000). Plasma concentration of asymmetric dimethylarginine, an endogenous inhibitor of nitric oxide synthase, is elevated in monkeys with hyperhomocyst(e)inemia or hypercholesterolemia. Arterioscler. Thromb. Vasc. Biol..

[29] Betz B., Moller-Ehrlich K., Kress T., Kniepert J., Schwedhelm E., Böger R.H., Wanner C., Sauvant C., Schneider R. (2013). Increased symmetrical dimethylarginine in ischemic acute kidney injury as a causative factor of renal l-arginine deficiency. Transl. Res..

[30] Ivashchenko C.Y., Bradley B.T., Ao Z., Leiper J., Vallance P., Johns D.G. (2010). Regulation of the ADMA-DDAH system in endothelial cells: A novel mechanism for the sterol response element binding proteins, SREBP1c and -2. Am. J. Physiol. Heart Circ. Physiol..

[31] Li J., Xia W., Feng W., Qu X. (2012). Effects of rosuvastatin on serum asymmetric dimethylarginine levels and atrial structural remodeling in atrial fibrillation dogs. Pacing Clin. Electrophysiol..

[32] Vladimirova-Kitova L.G., Deneva-Koycheva T.I. (2012). The effect of simvastatin on asymmetric dimethylarginine and flow-mediated vasodilation after optimizing the LDL level: A randomized, placebo-controlled study. Vascul. Pharmacol..

[33] Vladimirova-Kitova L.G., Deneva T.I. (2010). Simvastatin and asymmetric dimethylarginine-homocysteine metabolic pathways in patients with newly detected severe hypercholesterolemia. Clin. Lab..

[34] Xia W., Yin Z., Li J., Song Y., Qu X. (2009). Effects of rosuvastatin on asymmetric dimethylarginine levels and early atrial fibrillation recurrence after electrical cardioversion. Pacing Clin. Electrophysiol..

[35] Oguz A., Uzunlulu M. (2008). Short term fluvastatin treatment lowers serum asymmetric dimethylarginine levels in patients with metabolic syndrome. Int. Heart J..

[36] Sicard P., Delemasure S., Korandji C., Segueira-Le Grand A., Lauzier B., Guilland J.C., Duvillard L., Zeller M., Cottin Y., Vergely C. (2008). Anti-hypertensive effects of Rosuvastatin are associated with decreased inflammation and oxidative stress markers in hypertensive rats. Free Radic. Res..

[37] Tanaka N., Katayama Y., Katsumata T., Otori T., Nishiyama Y. (2007). Effects of long-term administration of HMG-CoA reductase inhibitor, atorvastatin, on stroke events and local cerebral blood flow in stroke-prone spontaneously hypertensive rats. Brain Res..

[38] Chen P., Xia K., Zhao Z., Deng X., Yang T. (2012). Atorvastatin modulates the DDAH1/ADMA system in high-fat diet-induced insulin-resistant rats with endothelial dysfunction. Vasc. Med..

[39] Nishiyama Y., Ueda M., Otsuka T., Katsura K., Abe A., Nagayama H., Katayama Y. (2011). Statin treatment decreased serum asymmetric dimethylarginine (ADMA) levels in ischemic stroke patients. J. Atheroscler. Thromb..

[40] Lu T.M., Ding Y.A., Leu H.B., Yin W.H., Sheu W.H., Chu K.M. (2004). Effect of rosuvastatin on plasma levels of asymmetric dimethylarginine in patients with hypercholesterolemia. Am. J. Cardiol..

[41] Eid H.M., Eritsland J., Larsen J., Arnesen H., Seljeflot I. (2003). Increased levels of asymmetric dimethylarginine in populations at risk for atherosclerotic disease. Effects of pravastatin. Atherosclerosis.

[42] Young J.M., Strey C.H., George P.M., Florkowski C.M., Sies C.W., Frampton C.M., Scott R.S. (2008). Effect of atorvastatin on plasma levels of asymmetric dimethylarginine in patients with non-ischaemic heart failure. Eur. J. Heart Fail..

[43] Valkonen V.P., Laakso J., Paiva H., Lehtimäki T., Lakka T.A., Isomustajärvi M., Ruokonen I., Salonen J.T., Laaksonen R. (2003). Asymmetrical dimethylarginine (ADMA) and risk of acute coronary events. Does statin treatment influence plasma ADMA levels?. Atheroscler. Suppl..

[44] Paiva H., Laakso J., Lehtimaki T., Isomustajarvi M., Ruokonen I., Laaksonen R. (2003). Effect of high-dose statin treatment on plasma concentrations of endogenous nitric oxide synthase inhibitors. J. Cardiovasc. Pharmacol..

[45] Laufs U. (2003). Beyond lipid-lowering: Effects of statins on endothelial nitric oxide. Eur. J. Clin. Pharmacol..

[46] John S., Schneider M.P., Delles C., Jacobi J., Schmieder R.E. (2005). Lipid-independent effects of statins on endothelial function and bioavailability of nitric oxide in hypercholesterolemic patients. Am. Heart J..

[47] Maeso R., Aragoncillo P., Navarro-Cid J., Ruilope L.M., Diaz C., Hernández G., Lahera V., Cachofeiro V. (2000). Effect of atorvastatin on endothelium-dependent constrictor factors in dyslipidemic rabbits. Gen. Pharmacol..

[48] Neveux N., David P., Cynober L., Cynober L. (2004). Measurement ofamino acid concentrations in biological fluids using ion exchange chromatography. Metabolic and Therapeutic Aspects of Amino Acids in Clinical Nutrition.

[49] Ricart-Jane D., Llobera M., Lopez-Tejero M.D. (2002). Anticoagulants and other preanalytical factors interfere in plasma nitrate/nitrite quantification by the Griess method. Nitric Oxide.

[50] Allain C.C., Poon L.S., Chan C.S., Richmond W., Fu P.C. (1974). Enzymatic determination of total serum cholesterol. Clin. Chem..

